# Weight stigma experiences and self-exclusion from sport and exercise settings among people with obesity

**DOI:** 10.1186/s12889-021-10565-7

**Published:** 2021-03-22

**Authors:** Hendrik K. Thedinga, Roman Zehl, Ansgar Thiel

**Affiliations:** 1 Institute of Sport Science, Eberhard Karls University Tübingen, Tübingen, Germany; 2 Interfaculty Research Institute for Sport and Physical Activity, Eberhard Karls University Tübingen, Tübingen, Germany; 3 Institute of Sport Science, University of Bern, Bern, Switzerland

**Keywords:** Obesity, Physical activity behaviour, Sport and exercise settings, Weight stigma, Discrimination, Coping, Self-exclusion, Social withdrawal

## Abstract

**Background:**

A central strategy to tackle the health risks of obesity is regular physical activity (PA), exercising and participating in sports. However, people with obesity regularly experience weight-related stigma and discrimination in sport and exercise settings. Research has indicated that they often cope with such experiences by simply excluding themselves from sport and exercise. Meanwhile, self-exclusion as a coping strategy has not been fully understood and it remains unclear to what extent self-exclusion from PA settings is accompanied by general inactivity among people with obesity. The goal of this interview study was to determine to what extent physical inactivity among adults with obesity is the result of weight stigma-induced self-exclusion in and from sport and PA settings.

**Methods:**

We conducted semi-structured interviews with thirty adult men and women with obesity (average BMI: 40.64) and asked them about experiences with their body, weight stigma and coping behaviours in sport and exercise settings across their lifespans. Employing constant comparative analysis and a thematic network approach, we analysed the interview data to identify the most common reasons for and different strategies of self-exclusion.

**Results:**

Participants reported that they excluded themselves from sport and exercise settings due to traumatic weight stigma experiences, self-discrimination and fear of stigma, using a variety of strategies. Exposure to discrimination was prevented by selectively avoiding certain settings or strategically frequenting them at certain times only, but also by exercising in ‘safe’ spaces, e.g. at home. Furthermore, people with obesity reported strategically managing their social relations in order to avoid stigmatising reactions by others in exercise settings, for example by exercising individually and avoiding social PA. Most notably, our results strongly indicate that not all self-excluding coping strategies result in less exercising.

**Conclusions:**

In order to successfully promote physical activity among people with obesity, the various forms of self-exclusion should be taken into account as pathways of stigma regarding physical activity. People with obesity may exclude themselves from certain PA settings, yet could still be exercising on their own or in other ways. One focus of public health strategies should thus be directed at the potentially socially isolating effects.

**Supplementary Information:**

The online version contains supplementary material available at 10.1186/s12889-021-10565-7.

## Background

Obesity is one of the major public health concerns worldwide [1]. A key strategy to reduce the associated health risks of obesity is regular physical activity [1] exercising and engaging sports. However, people with obesity^1^ frequently experience weight-based stigma in physical activity related settings such as sport^2^ and exercise settings [3]. Since the promotion of sport, exercise and physical activity programmes for people with obesity represents a crucial task for public health initiatives, these barriers require further investigation.

### Weight stigma as a barrier to exercise and physical activity

In the last decade, researchers have increasingly investigated weight stigma as a potential barrier to physical activity and exercise [4]. A stigma refers to “an attribute that is deeply discrediting” ([5] p.13) to a person’s social identity according to Goffman’s widely known definition [5]. In a more recent concept, Link and Phelan [6], conceptualise stigmatisation as a process including five components: firstly, a certain characteristic, such as higher body weight or obesity, is identified and labelled as deviant from a constructed norm. This process is called labelling and it describes how humans categorise differences [6]. The next component of Link and Phelan’s concept is stereotyping [6]: as a result of being categorised as different and deviant, a labelled person gets pigeonholed by linking him or her to the group with same deviant characteristic [6, 7]. Stereotypical beliefs that exist about this deviant group, for instance about people with higher body weight, get activated and are linked to the labelled person [6, 8]. Subsequently, groups which are associated with negative stereotypes become treated as separate from the so called normal group. The separated group is regarded as undesirable and even dangerous to some extent. The labelled, stereotyped and separated group therefore experience status loss and discrimination [9]. Finally, stigma processes take place in conditions of power; this means that stigma occurs only if powerful groups and interests reproduce this labelling, stereotyping, separation and discrimination [6, 9].

In general, discrimination refers to the “disadvantageous differentiation of individuals or groups” ([9] p.388). A person may for instance experience discrimination by being treated unfairly in a job application [9, 10]. More regularly, “day-to-day discrimination [ …] comes in the form of “micro-aggressions” such as […] misguided comments” (11 p.n.p.), rude and offensive remarks [9]. Discrimination represents a critical component in the stigmatisation process with regard to how “labelling and stereotyping lead to social inequalities” ([6] p.372). Furthermore, discriminatory behaviours are oftentimes overt forms of behaviours [6] through which the stigmatised person experiences social exclusion and devaluation. However, Link and Phelan strongly emphasize that no all forms of discrimination are clearly noticeable behaviours [6] but that they can be more “insidious” ([8] p.528).

Oriented towards the classification system by Lewis et al. [11], based on the work of Link and Phelan [8], different types of discriminatory experiences can be distinguished [9]: *overt or direct* discriminatory experiences (e.g. verbal abuse of people with obesity); *indirect* forms of discriminatory experiences (e.g. perceptions of being stared at judgingly), *environmental* discrimination (e.g. air plane seats that are too small), and *fear of* discrimination as a further form of stigma (e.g. voicing the anticipated fear of becoming a victim of disrespectful comments).

A further important form, not explicitly named by Link and Phelan and Lewis et al., is *self-discrimination*. Goffman already highlighted the relationship between “other persons’ concerns and definitions regarding the individual whose identity is in question” ([5] p.129) and, on the other hand, the stigmatised *own* subjective definition of his or her identity [5]. Perceiving and experiencing stigma by others may lead to the individual seeing him or herself in stigmatising terms [5]. Corrigan’s widely used term for this is self-stigma [12] and it occurs when stigmatised people agree with negative stereotypes and internalise them [12]. For example, “persons with overweight and obesity report sharing society’s biased attitudes toward excess weight, believing that they, in fact, are lazy, undisciplined, or otherwise undesirable because of their weight” ([13] p. 2). Corrigan predicts that this self-stigma and self-discrimination may lead to negative behavioural outcomes which “will significantly interfere with a person’s life goals and quality of life” ([12] p. 38).

Having a slim or athletic body and a fit appearance is conventionally regarded as healthy, while the obese body is commonly seen as highly deviant and unfit [9, 14]. The obese body and fatness are stereotypically associated with laziness, lack of will-power and lack of discipline [15, 16] because of the widespread societal belief that people with obesity simply overeat and do not exercise enough [15, 16]. Health and sports experts and the news media in general often frame obesity as a severe public health threat, the so called ‘obesity epidemic’ [17], and furthermore “focus on personal responsibility” ([17] p. 952) blaming the individual with obesity for not leading a more healthy lifestyle [17]. This leads to a separation and having an obese body therefore means to have a social status loss [9]. Accordingly, many people with obesity experience weight stigma and discrimination almost every day in many social sectors [17]. They regularly encounter “negative weight-related attitudes and beliefs demonstrated by stereotypes, rejection and prejudice” ([16] p.347). In this regard, sports and exercise settings are particularly difficult [3] because in many societies the obese body has become a symbol of personal failure to be active and healthy [14].

Research indicates that weight stigma is gendered, meaning that women with obesity experience weight stigma differently than men [18]. A review by Fikkan and Rothblum [18] has shown that women with obesity experience more weight bias and discriminatory treatment than men in several social domains such as work, education, and romantic relationships [18]. In the context of dating, Saguy highlights that women with obesity experience specific forms of discrimination that men do not [19]. A possible explanation for this gendered nature of weight bias could be that beauty and body standards are even higher for women than they are for men [18, 19]. However, there is still a lack of stigma research focusing on gender [18]. This is also the case for health-related settings.

In general, the health-related consequences of experiencing weight stigma are extensive and severe, and they have been highlighted by a growing amount of research over the last decade [4, 20–22]. A recent systematic review by Wu et al. [23] illustrates that weight stigma has been linked to weight gain, risk for diabetes, heightened stress levels as well as to anxiety, eating disturbances and depression [23]. Based on similar findings, Puhl and Heuer [20] therefore state that “weight stigma poses a significant threat to psychological and physical health” ([20] p.1023). This highlights the urgency to investigate social settings where people with obesity may experience stigmatisation.

Sport- and exercise-related settings have a high potential for weight-related stigma and discrimination [3, 4, 24]. Several studies conducted with children and adolescents have established that stigma experiences such as weight-related teasing appear to correlate with less physical self-efficacy among students and decreased physical activity [4, 25, 26], “likely because they have learned that removing themselves from physical activity settings altogether is a “safer” option than participating in these activities and potentially being scrutinized and teased by peers” ([26] p.373).

Research on weight stigma and physical activity among adults is still scarce [4]. However, in the last decade a growing amount of studies have investigated exercise behaviours of people with obesity, including some qualitative studies analysing the experiences of athletes with obesity in sport and exercise settings [27, 28]. Despite frequent perceptions of marginalisation and omni-present negative messages, a study by Scott-Dixon [29] also highlighted that in strength and power-based sports and exercise environments, such as weight-lifting, size and strength are also perceived as advantageous. Nonetheless, the majority of works strongly emphasizes the negative impact of weight stigma [30–32].

Studies have shown that perceived weight discrimination and past experiences with teasing are associated with a desire to avoid exercising and less moderate and strenuous activities [33, 34] and an unwillingness to participate in physical activity [11, 35]. Besides overt discriminatory experiences, the awareness that obese bodies are framed as unhealthy and unfit in physical and exercise settings can even lead higher-weight people to avoid physical activities [36]. More recent research has focused particularly on the frequent problem of self-stigmatisation among individuals with obesity often self-stigmatise, i.e. internalising stigma and weight bias thus directing negative attitudes and beliefs against themselves [37]. These studies suggest that self-stigmatisation may also have a detrimental effect on exercise behaviours and attitudes [38–41].

### Coping with weight stigma and physical activity

The extent to which stigmatisation and discrimination have an effect on the willingness to be physically active depends on how people with obesity respond to and cope with weight stigma experiences [9], specifically those occurring when exercising [42, 43]. The significance of coping strategies for health behaviours in the stigma process has been indicated by in recent studies by Himmelstein, Puhl, and colleagues [42]. In general, stigma-related coping ‘techniques’ have the function to manage the ‘spoiled social identities’ of stigmatised people, according to Goffman [5]. In his fundamental work on stigma, he describes a multitude of specific strategies that stigmatised people use in different social situations in order to protect their discredited identity from further harm [5].

Coping strategies can thus help to moderate the negative consequences of stigma for an individual’s mental health, e.g. by seeking social support or professional help. With regard to physical activity, a study by Wilfey et al. [44] has shown that participants who had better scores at coping with teasing also had fewer barriers to physical activity.

Coping with stigma can also be ‘maladaptive’ [45] and have a negative impact if, for instance a person consequently engages in an unhealthy lifestyle and avoids exercising [42]. One specific coping strategy appears to be crucial with regard physical inactivity: withdrawal from or avoidance of potentially threatening situations [21, 46]. In a comprehensive theoretical model on stigma management, Meisenbach [47] predicts that stigmatised individuals “are also likely to *isolate themselves* from society” ([47] p.280) and furthermore names the strategy of avoiding specific situations [47]. As a significant consequence, these strategies of avoidance and withdrawal socially marginalise stigmatised people. Importantly, Meisenbach highlights that the stigmatised individual chooses him or herself to do so in order to protect his or her identity [47]. Cachay and Thiel [48] have referred to this kind of self-imposed reduced engagement and non-participation as ‘self-exclusion’. In contrast to ‘exclusion through others’, when individuals are denied access to social contexts through a number of different excluding mechanisms, the individuals exclude themselves despite formally having access to it [48]. This kind of withdrawal can range from partial (social) self-marginalisation in a certain setting to the complete avoidance of settings.

Studies on coping behaviour regarding obesity stigma are generally scarce [42, 49], particularly with regard to public sport and exercise-related situations [43], and even more so with regard to the complexity of the strategy of avoidance and social withdrawal as a response to obesity stigma experiences. Some studies focused on everyday social situations and occasionally on physical activity and exercise settings [11, 45]. To our knowledge, only few studies [3, 43, 50, 51] have analysed how exercise-related discriminatory experiences may lead to self-exclusion and social withdrawal from physical activity settings. An interesting assumption in this regard was stated by Vartanian and Novak [41], namely that stigmatised individuals may avoid public exercise settings, such as gyms, due to weight stigma, but may nonetheless exercise in other ways. A recent interview study with women with obesity by Myre, Glenn [43] indicated that individuals may cope with physical activity related stigma by changing their exercising behaviour in specific protective ways. Hence, it appears that the setting and the prospect of discrimination, not necessarily the activity itself, play a central role in whether individuals with obesity avoid physical activity and exercising, thus also suggesting that avoidance may be a far more complex behaviour. Vartanian and Novak therefore recommended future research to take into account that setting-specific experiences could be decisive for whether or not people with obesity withdraw from physical activity in order to cope with stigma [41]. This suggestion particularly makes sense when considering that existing questionnaires in quantitative studies have assessed exercise avoidance with only very few and broad items [41, 52].

Against this background, we decided to conduct a qualitative in-depth interview study with people with obesity about their body- and weight-related experiences in different sport and exercise settings across their life-spans.

Our study particularly investigates the question: to what extent is physical inactivity among adults with obesity the result of a weight stigma-induced self-exclusion in and from sport and PA settings?

Our study can therefore provide important insights into how people with obesity perceive weight stigma in sport and exercise settings and how they cope and react to it.

## Methods

### Approach

The data presented here is part of a larger mixed-method interview study conducted with 30 women and men with obesity in Germany [9]. The study’s goal was to explore the participants’ experiences, attitudes, and perceptions on a wide range of body-related issues in several social contexts [9]. Our analysis is based on a social constructivist paradigm. From this perspective, social reality is not objectively pre-defined but gets meaning through interpretation and the assignment of meaning [53, 54]. We conducted semi-structured interviews employing a biographical approach – giving participants the opportunity to talk about their body and weight-related experiences across their life spans. The interviews covered a broad scope of health and body-related topics and issues. Biographical mapping data of this study has already been published [9]. One of the interviews’ major foci was on the participants specifically describing their coping responses to weight-based stigma in sports and exercise across their life spans. This paper presents the qualitative data from these interviews regarding this subject.

### Recruitment of participants and sample

Since previous research on weight stigma has predominantly focused on children and (younger) female adults [4], we chose to interview adults with obesity and to include both male and female participants. Our intention was to interview a broad variety of people (in a relationship, single, with and without children, with different occupation and of different age groups) [9]. All participants were to have a BMI of at least 30, what is regarded as obesity [9, 55].

Firstly, we recruited participants by asking patients from the Tübingen University Hospital Help Centre for the treatment of patients with morbid obesity [9]. Secondly, we handed out flyers at physicians’ offices, gyms, physiotherapy practices, community schools and colleges in the wider area of Tübingen and Stuttgart [9]. Thirdly, we emailed all members of the University of Tübingen via a central mailing list [9]. Flyers and mails informed readers that we were seeking people with obesity to participate in a study about stigmatisation and prejudices in exercising and sport contexts. Interviewees were to receive a compensation of EUR 50 each [9].

We managed to recruit 30 people (14 men and 16 women) with obesity (average BMI: 40.64 and average age 37.66 years) from the area of Tübingen and Stuttgart, southwest Germany.

More details regarding the participants’ occupations and relationship statuses can be found in a Table 1 which we previously published [9].

**Table 1:** General characteristics of recruited sample [9]

**Gender,** ***n***	
Male	14
Female	16
**Age (span),** ***n***	
25–29	4
30–39	16
40–49	5
50–59	4
**Age (mean),** ***years***	37.66
**BMI,** ***kg/m***^***2***^	
Range	33–58
Mean	40.64
**Occupation,** ***n***	
Fulltime student	6
Student with job	4
Academic	4
Printing job	1
Police officer	1
Sales person	2
Driving instructor	1
Sound engineer	1
Nurse	1
Industrial mechanic	1
Office assistant	2
Bus driver	1
Administration secretary	1
Unemployed	4
**Partner status,** ***n***	
Single / or no current relationship	15
Married / or in a relationship	13
Divorced	2
**Children,** ***n***	
Children	9
No children	21

We began initial analysis during the data collection period, discussing participants’ experiences and emerging themes with all interviewers. This was done in iterative steps. At a certain point it became clear that participants kept reporting similar experiences. Since we had already gathered a substantial amount of interview data from thirty people, we did not recruit more participants because in our estimation no significantly new themes would emerge. This decision was thus also made for research-economic reasons because we felt that the saturation of data already achieved did not justify the additional research costs of the execution and analysis of more interviews.

### Execution of the interviews

We conducted the interviews face to face. Since body-related discriminatory experiences are often considered a sensitive topic, with participants with obesity potentially feeling embarrassed about it, especially when interviewed by the opposite gender [56], we decided that all participants were to be interviewed by people of the same gender. To this end, we recruited several interview assistants from our institute [9] whom we extensively trained in weekly meetings over a period of 2 months on the topics of obesity, stigmatisation and physical activity. The training furthermore covered qualitative methods and the process specific to our semi-structured interviews and interview guide, and integrally included mock-interviews with extensive feedback [9]. Male participants were interviewed by author (HKT) and additional male assistants; female participants were interviewed by trained female assistants. The interviews were conducted between June 2015 and February 2016. Each interview lasted 90 min on average [9].

Supervised by author (AT), author (HKT) and author (RZ), all interview assistants met on a weekly basis for counselling and to discuss the execution of the interviews. Collecting individual feedback from the interviewers in order to monitor and analyse process and – when necessary – modify questions was integral to these sessions.

We decided on semi-structured interviews to enable participants to openly talk about their experiences [57]. In contrast to a strict questionnaire protocol, semi-structured interviews allow for a natural conversation to unfold, allowing interviewees to open up, share personal stories, and ‘explore sensitive topics’ [58].

We structured the interview into six main thematic blocks which covered a range of health and body-related topics, featuring several open questions respectively to initiate discussions. The interview question guide was designed using a biographical approach, inviting participants to individually reflect on, share and discuss perceptions and experiences across their life span.

The data analysed for this paper presents exercise histories, specifically experiences of and coping with stigma in sport and exercise settings. Some of the questions employed were based on a set of questions proposed by Lewis et al. [56]. Yet, it is important to note that we aimed to differentiate between ‘general stigma’ and ‘exercise-related stigma’. A selection of exemplary central questions is listed in Table 2.

**Table 2:** Exemplary central questions

Topic: Exercise history, experiences of and coping with weight stigma in sport and exercise settings	Central question(s)
Attitude towards sport and exercise	What is your attitude towards sports and exercise? Are you interested in sport or exercise? Why, why not?
Sport and exercise history	How would you describe your sport and exercise activities across your life span? Were there any times when you were doing a lot of/or almost no exercise or sports?
(Coping)	What were the reasons you stopped?
	Are you currently doing any sports or exercise? Why, why not?
Stigma experiences in sport and exercise settings	What positive and negative experiences have you had in sport/when exercising? Did you ever feel disadvantaged or discriminated against or confronted with prejudices? Can you recall any specific experiences? What experiences did you have during PE classes at school/ or sports clubs?
Coping	How did that make you feel? How did you respond?

### Data interpretation

All interviews were audiotaped and transcribed verbatim following modified transcription rules proposed by Lamnek and Krell [54]. All interviews (with one exception) were held and transcribed in German. One interview was conducted and transcribed in English because the participant was English-speaking. Relevant participants’ quotes used for this publication were translated from German into English by the authors and proofread by a professional language editor who is a native speaker in both English and German.

In order to interpret the interview data, we used a thematic network analysis [59]. Data interpretation was led by author (HKT) and author (AT). Initial discussion of the data took place during the data collection period. The main systematic analysis took place after all interviews had been conducted and transcribed, and it comprised several steps. For this, authors (HKT) and (AT) held weekly meetings with assistants. The first steps included readings and re-readings of transcripts while coding the data. For the purpose of the overall interview study, each interview was analysed inductively with regard to several main thematic units.

For the purpose of this paper, authors (HKT) and (RZ) re-read and analysed every interview once more to identify all passages in the data concerning self-exclusion and self-inclusion in and from sport and PA settings. Our analysis primarily focused on self-exclusion; however, this also included viewing stories of self-inclusion, for instance when participants did not completely avoid exercise and physical activity. Employing constant comparative analysis, we then organised the data by identifying recurrent basic themes [60, 61] concerning the questions of why and how participants excluded themselves from sport settings. The particular focus was on perceptions and management of weight stigma. During this process, emerging ‘basic themes’ in the data and corresponding transcript passages were frequently discussed by all three authors in debriefing sessions; themes were also compared with initial analyses of each interview. Subsequently, the basic themes were summarised by more abstract ‘organising themes’ [60, 61]. In the next step, basic and organising themes were discussed by all authors in order to reach agreement on the main thematic groups in the interview data. This way of organising the data is based on the work on thematic networks by Attride-Stirling [59]; the main themes were therefore regarded as ‘global themes’ which sum up, condense and include all key aspects of organising and basic themes (see also [61]). In a final step, we defined five global themes in order to highlight the main findings in the data with regards to why and how participants employed self-excluding coping strategies in sport settings.

### Statement on positionality of research team

Concerning the research team’s positionality, we would like to explicate that all authors are sport scientists who share a life-long history of participating in sports and exercising. Author (HKT) also recently started working as a teacher. With regard to body and the issue of insider vs. outsider perspective [22], none of the authors would self-identify as overweight. We have therefore not experienced weight stigmatisation ourselves. However, the team has a research history regarding weight stigma and published several studies on the subject [9, 14, 62]. Consequently, we are familiar with the subject matter [22].

## Results

**Fig. 1:**
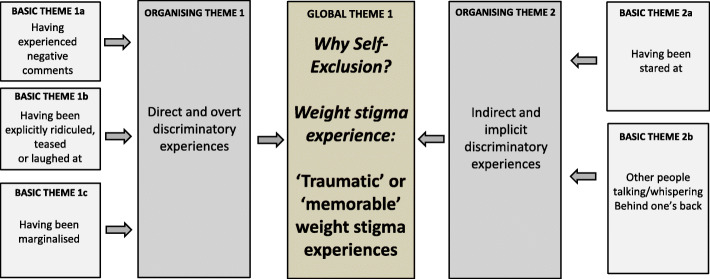
Thematic Network 1 (Reasons for Self-Exclusion): Traumatic or memorable weight stigma experiences

**Fig. 2:**
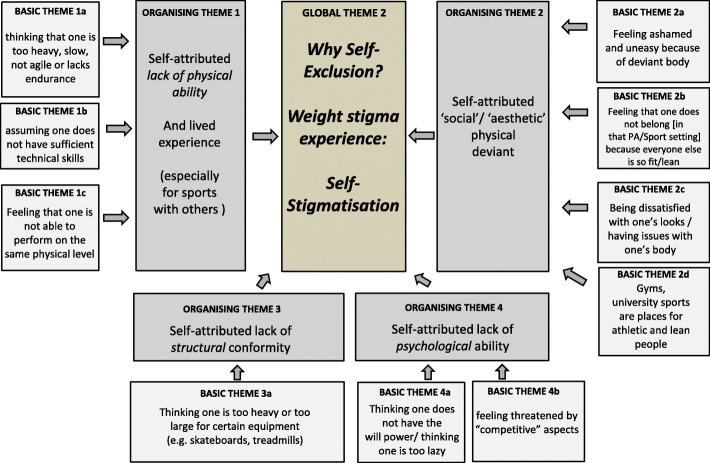
Thematic Network 2 (Reasons for Self-Exclusion):  Self-stigma

**Fig. 3:**
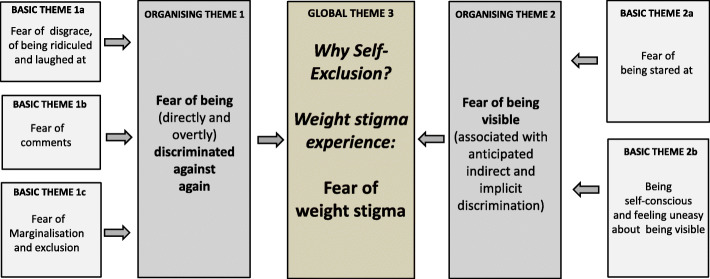
Thematic Network 3 (Reasons for Self-Exclusion): Fear of weight stigma

**Fig. 4:**
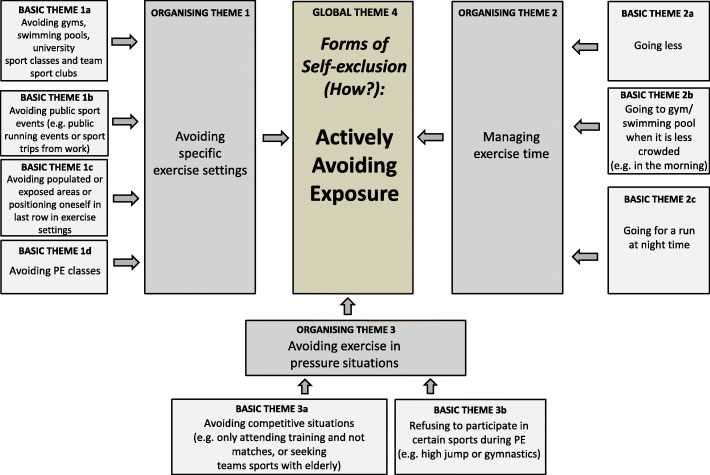
Thematic Network 4 (Strategies of Self-Exclusion): Actively avoiding exposure

**Fig. 5:**
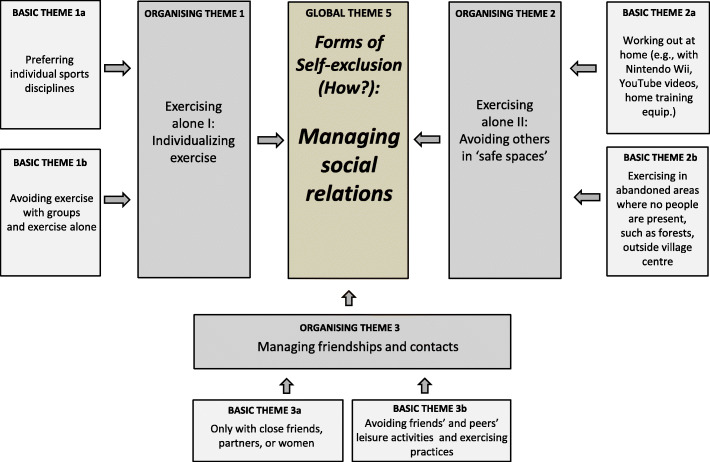
Thematic Network 5 (Strategies of Self-Exclusion): Managing social relations

With regard to weight stigma experiences, five global themes became evident in the data concerning the pathways of self-exclusion of people with obesity from sport and exercise-related contexts. Regarding the question of ***why*** people with obesity exclude themselves, we identified three main reasons: 1) ‘Traumatic’ or ‘memorable’ weight stigma experiences; 2) Self-stigmatisation; 3) Fear of weight stigma. With regard to ***how*** people with obesity exclude themselves, we found two key strategies: 4) Actively avoiding exposure; 5) Managing social relations. For a better overview of findings, each global theme including its organising and all basic themes are presented in Figs. 1, 2, 3, 4, and 5 at the beginning of each result section. The design of Figs. 1, 2, 3, 4, and 5 was inspired by Thomas et al. [61] and is based on our former collaborative work on a study [60]. In order to support the themes, we will present excerpts from the transcripts in each section. These citations were translated from German into English by author HKT, who holds a degree in English. Furthermore, translations were proofread by a professional language editor who is a native speaker in both English and German.

### Reasons for self-exclusion

The following themes and data excerpts were identified regarding the question ***why*** participants excluded themselves:

#### Global theme one: ‘traumatic’ or ‘memorable’ weight stigma experiences

Participants frequently mentioned and described upsetting discriminatory experiences as a reason to withdraw from and avoid sport and exercise. Such incidents could occur throughout the participants’ lifetime. We identified two main organising themes in this regard. Firstly, participants reported that they had had very upsetting ***‘overt’ and ‘direct’ discriminatory experiences*** in sport and exercise settings which led to their withdrawal from and avoidance of that exercise setting and deterred them from returning. These overt discriminatory experiences included negative comments and insults. The experience of being teased or laughed at during PE classes by peers which led to such frustration and sadness that participants lost interest in participating in PE, was common. Participant 19 (male, 65 y/o) described his reactions to such traumatic experiences in PE at school:

“Sometimes I just felt like crying. Because sometimes, well, absolutely … not good … When you always have this constant frustration during PE class, you eventually end up not doing it at all. Eventually you’ll sit down on the bench and say ‘guys, just leave me alone.”

Importantly, direct discriminatory comments were also reported to have been uttered by from teachers and coaches. Participant 29 (female, 33 y/o) reported the following incident with her coach as the reason for her leaving the gymnastics club as a teenager:

“P: She approached me and told an eleven-year-old child ‘you are the best dancer here but it simply doesn’t look nice with your weight’.

I: Ok, how did you react at the time, how did you cope with that at the time?

P: I was completely dumbfounded … um … I asked her whether she was serious and she said yes, and then I said that I’m not coming anymore, and then she followed me and said that I had misunderstood her, and then I told her I didn’t want to hear anything anymore, and still in front of her … I burst into tears in front of her.”

Furthermore, overt traumatic discriminatory experiences included incidents of participants being marginalised in sport and exercise settings. Common reports addressed experiences from their teenage years where they were picked last for games during PE classes or were ‘benched’ by sports club coaches during important games or tournaments. Some participants reported that they never partook in those team sports again due to these experiences. In a few cases, participants stated that gym trainers had unmistakeably suggested the gym was not the right place for people without discipline. In these cases, participants were assumed to lack discipline because of their body size:

“Every time I went to the gym, there was discussion with him [the gym trainer], and he pretty openly commented that he did not believe I had the discipline to last there in the long run. [ …] he only did that to me. And I was the only person in that place who was overweight and went there on a regular basis” (P. 2 male, 28 y/o).

Trainers reportedly even told them that other members had made complaints about ‘their smell’. This gave them the strong impression that they ‘were not welcome’, and as a reaction they left.

As the second organising theme, we identified experiences of ***indirect and subtle (*i.e.** ***less overt forms of) discriminations***. Participants reported situations in sport and exercise settings where they had been stared at with disdainful and judgemental glares. A further basic theme we identified describes incidents where people had been ‘whispering’/gossiping behind one’s back or at a distance. ‘Gossiping’ football moms were mentioned in this context.

#### Global theme 2: self-stigma

Self-exclusion from sport and exercise settings among people with obesity is often related to self-stigma. Our interviewees frequently expressed strong self-discriminatory attitudes towards their physical appearance and abilities. As the two most common forms of self-discrimination in sport and exercise settings, we identified ‘self-attributed lack of physical ability’ and ‘self-attributed social/aesthetic deviance’. ***Self-attributed lack of physical ability*** means that participants described themselves as being too slow, lacking endurance, or lacking technical skills to do sports with friends or others in a variety of situations. Describing this perception of physical inferiority, participants often were in fact describing their lived experiences of being active with less mobile bodies:

“So all these ball and group games and so on, I really didn’t like them. Simply because of the weight, I was for a long … extremely slow.” (P. 11, female, 30 y/o)

However, experiences like these caused them to feel too slow even for sports they had not tried out yet:

“Yes, I think at least a few years ago I would have liked to play basketball because that always interested me but ( … ) I would have to lose some serious weight to be able to play one of the faster positions.“ (P16, male, 30 y/o)

Both the lived experience and perception of physical inferiority had thus played an important role for participants to leave and avoid team sports across their life-spans. Participants also mentioned the fear of being a burden to the team in this context.

In contrast to concerns about the *ability* to physically perform, ***self-attributed social/aesthetic deviance*** focused on perceiving one’s larger body as *looking* unnatural and peculiar. Participants stated that they had been concerned with feeling abnormal in sport and physical activity settings. In this regard, participants explained for example that they did not belong and felt alien in settings where everyone else was so lean and good-looking:

“It really feels like you’re someone from the outside here, a foreigner or, yes, some person from Mars.” (P. 24, female, 43 y/o)

Dissatisfaction with looks and bodies played an important role in this regard. Participants furthermore described many sport settings, especially gyms, sports clubs and university sports classes, as places for athletic and lean people.

“I think, um, in gyms it’s like that the lean and the athletic people are among themselves. If someone like me turned up, yes, well I mean most people don’t look like me. I would be someone who would stand out initially.” (P. 6, male, 33 y/o)

As a result, several participants explained they had felt ashamed of their overweight bodies in these settings, which often led to their withdrawing from them.

Less common forms of self-discriminatory attitudes among the participants were connected to ***environmental conformity*** and ***lack of psychological ability***. A few of the participants explained that they had been worried about being too heavy to use certain sports equipment such as treadmills. Furthermore, participants concurred with negative stereotypes and described themselves as being too lazy or lacking the will-power to participate in certain sport and exercise activities. In this regard, they had felt uncomfortable and intimidated by the ‘competitiveness’ of sport activities.

#### Global theme 3: fear of weight stigma

A third major reason why people excluded themselves from sport and exercise settings can be found in the fear of being exposed to weight stigma. In this regard, we found two main organising themes in the data: firstly, the ***fear of becoming a victim of direct and overt discrimination*** again and, ***secondly, the fear of one’s body being highly visible***. In the first case, the interviewees already had had traumatic experiences in sport and exercise setting due to explicit discrimination before they decided to avoid similar situations. For instance, participant 25 (male, 30 y/o) talked about why he had decided against participating in a skiing trip that his company had organised:

“Then you’ll get laughed at again ( … ) such a monster on such small skis, and I feel, although I would like to go skiing, with my colleagues and all, but I don’t want to. I don’t want to expose myself to that. Maybe it’ll be all right but I can’t, I can’t risk it.”

In the second case, the interviewees avoided sport and exercise settings because they anticipated being stared at. In this regard, several participants specifically stressed the uneasy and unpleasant feeling of being visible in exercise settings. Female participant 29 (33 y/o) on stares:

“P: I used to be a very enthusiastic swimmer, I really loved it but I haven’t been to the swimming pool in eight years.

I: Ok.

P: I simply don’t dare to go at all, um … anxious about comments, fear of stares [ …] I simply wouldn’t feel comfortable.

[She goes on to talk about stares at the gym:]

“It’s rather an atmosphere of feeling uneasy because you have to overcome yourself and go and you think ‘please, please no-one watch me”

Open spaces and the presence of mirrors in gyms were also mentioned in this regard.

### Strategies of self-exclusion

The following themes and data excerpts were identified regarding the question of ***how*** participants excluded themselves. Our analysis in the following sections focuses on different self-excluding strategies.

#### Global theme 4: actively avoiding exposure

As can be seen from Figs. 4 and 5 below, participants reported a multitude of different self-excluding coping strategies in order to deal with weight stigma in exercise and sport. To simply stop exercising altogether or for phases of their life was, of course, the most radical self-excluding strategy. And it must be noted that this was frequently mentioned. However, our results show that strategies of self-exclusion were often more subtle and did not necessarily mean that the participants completely avoided exercising.

Some participants only ***avoided specific settings***, often gyms and swimming pools, in which they particularly expected weight-related discrimination. Of significance in this regard is the finding that many participants had visited these settings for a while before they stopped. For instance, participant 27 (female, 44 y/o) replied to being asked whether she had noticed any reactions towards her body:

“I went the gym for two years, it made me feel uncomfortable. I cancelled [the membership] eventually. I went there because of my slipped disc. For two years I went regularly, two or three times per week, but then (-) I simply didn’t feel comfortable. That was, for me that was too, only obligation and no fun. So, I think with that you notice, so I also felt uncomfortable because of stares there” (our insertion).

Due to a number of different discriminatory experiences, many of the participants named gyms as a setting they hated or feared and thus specifically avoided. The same aversion was also strongly expressed towards swimming pools. Some of the participants reported phases in their lives when they refrained from trying to go at all:

“NEVER would I have dared to go outside, for God’s sake, to dare to go the swimming pool, pff … that wasn’t even a question.” (P. 17, female, 27 y/o)

Further settings the participants strategically avoided were university sports classes and sports clubs, specifically for team sports.

Participants also ***avoided the setting of ‘public sports events’*** such as public running events.

As a further strategy we identified the strategy to ***avoid populated or exposed areas*** in the above mentioned settings when exercising. This strategy was particularly used in gyms. For instance, participant 25 (male, 30 y/o) stated:

“I consciously avoided the whole dumbbells area and everything because that’s where the people, the athletic people, people with a good figure are. Um … there and um - doesn’t look that good when someone like [me] comes and – totally stomping as if he’s like – well like I said”

A further mentioned strategy was to position oneself in the last row at exercise classes.

PE classes were mentioned often as a setting where participants had felt exposed. Many participants had experienced weigh stigma there frequently. Due to the fact that bodily exposure cannot be avoided in PE classes, several participants tried to avoid attending PE as much as possible when attending school often pretending to be sick or otherwise unable to participate. Some female participants mentioned that they had pretended to be on their period in order to be excused from PE. Furthermore, both male and female participants stated that they had forged letters from their parents to be relieved from PE. Participant 25 (male, 30 y/o) told us the following about his experiences with his excess weight during PE at school:

“P: I never NEVER wanted to go to swimming lessons, I forged … um … letters to excuse myself as if they were from my parents.... that, that.

I: And why did you not want to?

P: Well … because they looked at me, because they laughed at me, the children. Children can be horrible. They laughed at me, they … um … bullied me [ … ]

… Well you got bullied at school, you (.) like I said, I forged letters to be excused from PE, that I’m sick or something, so I didn’t have to go. When I did attend PE class, I often … um … forgot my [sports] shoes (.) on purpose, so I could sit on the bench or something.”

Another relevant strategy to actively avoid exposure to stigma in exercise settings was ***‘managing exercise time’.*** The simplest strategy was to spend as little time as possible at exercise and sport settings. Some participants also stated frequenting certain exercise facilities, specifically gyms and swimming pools, when they were less crowded. Swimming pools (especially in the open air) were sought out in the early mornings only, when there were hardly any other people present. Finally, some male participants managed exposure (and therefore weight stigma) by going for runs in the evening or even at night-time only. Participant 16 (male, 30 y/o) thus stated,

“P: When I go for a run I usually go in the evening [ …] around nine, half past ten. I live in a village near a town [ … ]: After eight there is no one outside who could see you.”

I: Would that play a role for you that … um … people could see you?

P: Um, if I’m out of shape and starting again, yes!”

Finally, some participants specifically ***avoided ‘exercising in pressure situations’.*** One interesting result in this context was that participants reported about attending team training sessions in various sports, yet not attending or participating in the matches. Another strategy to avoid pressure was to choose team sports with elderly people, or, if possible, to avoid only certain sports with a high risk of body-related embarrassment during PE classes, such as high jump or gymnastics. These strategies highlight once more that participants did not necessarily completely avoid exercising after experiencing stigmatisation and discrimination.

#### Global theme 5: managing social relations

Our data so far already suggests that self-excluding coping responses do not necessarily lead to general exercise avoidance. Instead, people with obesity appear to manage exercise time, space, and situations in order to find ‘safe’ weight stigma free spaces. The interviews also illustrate that the participants coped with weight stigma by ‘managing’ their activity-related social networks. Typical strategies included avoiding ‘social exercising’ or to selecting ‘non-threatening’ partners for their activities.

As one important organising theme we identified that participants ***‘individualised’ exercise behaviour***. We asked participants directly how experiences of weight discrimination affected their physical activity behaviour. The answer by participant 2 illustrates this theme (male, 28 y/o):

“Nowadays I prefer individual sports. I swim on my own. So what I mean [ …] I mean that I swim my laps - even when I’m with a group - on my own. And I also do winter sports, which for me, so I also regard that as more of an individual sport, too.”

The self-excluding mechanism in this context becomes evident when considering that several participants stated they had practised team sports and numerous physical activities with peers in their childhood and adolescence. They also claimed to still be very interested in team sports and missing the fun of playing football in a team and exercising in groups. However, in order to avoid weight stigma, they mostly stopped engaging in team sports as adults and switched to individual sport activities.

In addition to weight stigma, independency, especially from competitiveness and other individuals’ physical superiority were named as reasons for the individualising of exercise. With further probing, however, participants emphasised their discomfort with exercising in groups rather than their physical ability. Avoiding groups was mentioned by particularly women, such as a female participant 14 (33 y/o):

“It used to be a bigger burden. Um … and the negative reactions … yes I believe this, this social pressure that you are simply exposed to as a student. Yes. And then during my time at university, I would have had the energy to participate in some class from university sports class, but for God’s sake – no! [ …] To imagine to [exercising] in a group of thirty people in one room – that’s a total nightmare for me. Instead, prefer the new Metallica album and [run] through the forest.’ (our insertion).

Another strategy to avoid such situations was to ***‘manage friendships and social contacts’*** by exercising *only* with very close friends or with partners. Furthermore, a strategy mentioned by female participants mentioned was to only exercise with other women and not in the presence of men, while the male participants avoided their friends’ leisure and exercising activities, such as informal football organised by fellow students.

As a final organising theme, we found that participants also ***exercised alone in ‘safe spaces’*** in order to completely avoid other people and withdraw from social and public spaces. Some also reported going for runs and bicycle tours in abandoned areas such as forests, or outside the town centre. Participants also mentioned that they worked out at home with home training equipment, online videos, or the Nintendo Wii. Participant 14 (female, 33 y/o) explained the safe feeling when exercising in this manner:

“You can just be by yourself on this ergo-bike; I can close my curtains and shut the door, and then I do it for myself and then it’s ok.”

Addressing this topic, the explained in some instances that they had quite intensively thought about how to create their own home training programme. Overall, this tendency to work out at home was mentioned more often by women.

## Discussion

Our study dealt with the extent to which physical inactivity in adults with obesity is the result of a weight stigma-induced self-exclusion from sport and PA settings.

Our results align with a number of studies which have shown that experiences of weight stigma over the lifespan are related to less activity in general and the avoidance of public sport and exercise facilities specifically [11, 21, 33, 34, 42]. Our results further illustrate that self-exclusion from sport and exercise settings is caused by a variety of weight stigma experiences, self-discriminatory attitudes, and anticipated fear of stigmatisation. Interestingly, participants did not link environmental exclusion to their own self-exclusion directly, though their accounts included mention of exclusionary physical environments. In accordance with findings by Lewis et al. [11], participants complained about gym equipment being designed only for normal-sized bodies, yet they did not mention this as a major reason for why they avoided the gym. This finding is in line with the results of a recent interview study according to which sports gear did not affect the willingness of female runners with obesity to exercise [51]. The only possible exception to this could be seen in reports about ‘perceived lack of structural conformity’ when participants *thought* that treadmills sounded too loud under their weight.

Instead, our findings suggest that self-exclusion from sport and exercise settings is to a large extent the result of ‘traumatic and memorable’ stigma experiences in similar settings across the life span. The social status loss inherent in stigmatisation occurs in many different social settings of everyday life for people with obesity [6, 21]. In this context, sport and exercise space represent settings of everyday life where weight stigma is particularly widespread because the body and normative fitness ideals play such a central role here [3]. From the perspective of those concerned, the obese body in sport and exercise settings is treated as highly deviant. Conversely, fit and athletic people have a higher degree of social power in these settings.

However, not only overt forms of discriminatory experiences, such as negative comments or ridicule, but also subtle ‘implicit’ discriminatory forms were perceived as traumatic by participants. The experience but also fear of disdainful stares in exercise settings also seems to be a factor in this context. This is in line with the wider prejudice literature where they are also known as micro-aggressions [63] In fact, a growing amount of research indicates that these so called “fat microaggressions” ([63] p.504) may be more harmful to a person’s health and health behaviours than overt forms of discrimination (see for example [63, 64]). The finding that such subtle forms of weight stigma may have a substantial impact on health behaviour is important to consider when designing future anti-discriminations initiatives and public health strategies. However, it also highlights how challenging such initiatives can be since it may be easier to change a coach’s or a health professional’s explicit comments than the way they ‘look’ at people with obesity.

Our results also support recent findings that the internalisation of weight stigma has an effect on exercise behaviour [38, 39, 41]. The interviews show that self-discriminatory attitudes concerning a variety of exercise-related physical and psychological skills (endurance, speed, agility, will power etc.) appear to be a reason for self-exclusion from sport and exercise settings. In this regard, we should stress that participants may have merely described their experience of exercising with obese bodies. Yet, we found evidence that participants held these attitudes also regarding activities they had not tried out yet. Furthermore, non-exercise-related self-discriminatory attitudes, such as a lack of self-worth or the self-description of their own bodies as highly deviant, were also reasons for self-exclusion, what is in accordance with Vartanian and Novak [41]. These findings support the recent call for more research on self-directed stigma and health outcomes [65].

Against the background of our biographical approach, it is important to briefly highlight the possible connections between the above findings. One could, for instance, argue that the repeated exposure to discriminatory incidents in physical activity spaces across their lifespan (overt verbal insults as well as subtle derogatory messages in the form of daily micro-aggressions) communicated to participants that they were not fit enough to exercise and did not belong in these settings. The self-stigma we found in participants’ stories may thus have been caused by the number of upsetting weight stigma experiences they remembered and reported.

Regarding the forms of self-exclusion, general avoidance of sports and exercise is a strategy that participants sought to prevent being discriminated against. Coping with weight stigma by avoiding physical activity might to a certain extent explain the negative health consequences of weight-related stigmatisation [42]. Yet, our results also strongly indicate that people with obesity do often not *just* generally avoid exercise or withdraw from sport settings, but exclude themselves from specific sport and activity settings, particularly those that threaten their self-esteem. A common strategy in this regard could be identified in the avoidance of gyms, swimming pools or PE classes. This goes in line with the few studies conducted on this topic [34, 56].

Our results also indicate that people with obesity do not necessarily exercise less as a response to weight stigma. Instead they appear to employ a multitude of strategies to be active in a way that allows them to avoid exposure to stigma. In this regard, they exclude themselves from certain situations but still exercise and participate and include themselves in certain sports activities. Vartanian and Novak [41] already hypothesised that people with obesity sometimes cope with weight stigma by strategically selecting their physical activities. Such strategies included avoiding certain (crowded) times at the gym or swimming pool, participating in training sessions only rather than also in competitions, or exercising strictly on by themselves. The latter strategy, e.g. running at night, had also been found by Inderstrodt-Stephens and Acharya [51]. Furthermore, a recent qualitative study conducted with women by Myre and colleagues [43] reported very similar results regarding altered physical activity, such as individual activity or avoiding busy times, in response to weight stigma in PA-related contexts. The finding that participants exercise more privately supports the Vartanian and Shaprow study [33] that found an impact of experienced stigma on moderate and strenuous activity but not necessarily on mild, total physical activity behaviour. Being active at home may include activities which are not as vigorous as those typically engaged in sport and exercise settings.

Our data thus shows that participants often sought ways to exercise on their own. From a health scientific perspective, being physically active alone is better than being physically inactive. The practise of avoiding *social* exercise furthermore allows people with obesity to avoid social situations in which lean and athletic people have the social ‘power’ to discriminate against them as the ‘bodily deviant’. However, self-exclusion from *social* exercising also has disadvantages. For people with obesity, social and emotional support from a strong social network is an essential factor regarding the sustainability of being active [21]. In this regard, team sport programmes appear particularly promising. Studies have shown that such programmes can have a greater positive effect on general fitness, self-esteem, and the motivation to be active in daily life, than standard exercise programmes [66, 67]. Faude et al. explain that “emotional support may be enhanced when sports is conducted together with peers, an effect likely to be more pronounced in team sports [ …] the social but also competitive nature of the game leading regularly to a feeling of success and coherence in the team” ([66] p.108). Social isolation and the lack of social support, in contrast, are strong predictors of bad health [68]. The dangers of social isolation as a result of coping with weight stigma have also been highlighted by Hunger et al. [21].

Finally we want to discuss some insights from this study regarding gender. In general, the interviews indicate that there are no stark differences when it comes to experiencing stigmatisation and discrimination in PA spaces. Both men and women described traumatic stigma experiences, self-stigma and reported fear of stigma when exercising. However, we should highlight that the quantitative results from our previously published biographical mappings [9] show that women on average remembered a stronger intensity of negative experiences in sports than men [9]. This indicates that, although both women and men are subject to discrimination in PA spaces, women may subjectively experience it differently. This none withstanding, it is noteworthy that both male and female participants felt very uncomfortable in gyms and public swimming pools and thus avoided these settings. This demonstrates how unwelcoming these environments must be for people with obesity of all genders.

With regard to the employed self-excluding strategies, there are a few points we want to furthermore highlight regarding gender. Participants stated that they went for runs in the evening or in abandoned areas. This would usually be regarded unsafe for women. Nonetheless, the interview study by Inderstrodt-Stephens and Acharya [51] with predominantly female runners with higher weight also showed that they resorted to running at night. Furthermore, the interviews indicate that women were particularly reluctant to exercise in larger groups and worked out at home. Against the background of the beneficial effects of exercising in groups, this should be addressed in public health strategies.

However, we can only tentatively highlight these subtle gender differences because it was not the primary focus of this analysis and because of the limited number of interviewed participants. Since the role of gender and other social categories still remains understudied in weight stigma research [18, 49] future studies should focus specifically on gender and investigate the various forms of self-excluding strategies with more participants.

Before addressing some practical implications of these results, we need to highlight and discuss some of this study’s limitations and strengths. Firstly, although we attempted to recruit participants from different social backgrounds with different occupations, a majority of our participants had an academic background, were either still enrolled as (part-time) students, or worked at the university. This, of course, limits the transferability of our results. Given that stigmatisation depends on the cultural and social context, these experiences may not be universal. In this regard, we must also point out the wide range in the participants’ BMIs. A person with a BMI of 33 may have other experiences than a person with a BMI of 45.

Secondly, we employed a number of different interviewers (for data collection). This decision was made because we could secure age- and gender-matched interviewer/participant combinations. On the one hand, this represents a strength of our study as it helped to foster a comfortable interview atmosphere for participants and it also ensured that we had several independent interviewers conducting the interviews. On the other hand, we cannot rule out that there may have been differences in the way certain interviewers phrased questions, responded to answers, and interacted with participants. Furthermore, it should be highlighted that the interviewers had bodies which would not be considered overweight. This body discordance between interviewers and participants may have also impacted the interview data we generated.

However, it should be mentioned that we did an extensive training of interviewers in weight stigma and interview execution (including practising the interview questionnaire several times) in order to overcome this. A further strength of the interview execution was that we encouraged participants to describe concrete and detailed examples of weight stigma experiences, also asking follow-up questions in order to encourage participants to clarify their statements [69]. Moreover, it is important to highlight some further strengths. In order to guarantee a high level of trustworthiness for this study, we adhered to several criteria proposed by Smith and Sparkes [70] and Korstjens and Moser [69]:

With regard to credibility, we adhered to “investigator triangulation” ([69] p.121) by having the researchers analyse the data independently. In this context, we also held regular peer debriefing sessions [70] with all authors in order to review and critically discuss the themes. We also observed the data persistently [69] by re-reading and re-analysing the data in detail several times. With regard to transferability [70], we described the context and execution of this interview study in as much detail as possible in the preceding sections.

### Practical implications for public health (educators) and recommendations for sport and exercise settings

Based on our findings, one could deduce that future public health strategies seeking to increase physical activity among people with obesity should primarily target their coping skills. However, such an individualised perspective bears the risk of neglecting that weight stigma is a complex social phenomenon firmly rooted in society. We therefore recommend an upstream approach of targeting and changing the social settings (specifically exercise and sport settings) in which stigma occurs. This will be a highly challenging task. Nonetheless, in the following we list a few practical implications and recommendations. Some of these suggestions have also been named by other experts [43, 46, 57]; for instance a recent work on body-inclusive spaces by Pickett and Cunningham [71] and an expert group consensus statement [72]:

- ***Educate Health Professionals*** (gym trainers, PE teachers, coaches, lecturers in health sciences at universities) about weight stigma and raise awareness for its potentially harmful effects. In this regard, we propose a mandatory training for PE teachers and any staff working in sport and exercise settings.
- ***More Inclusive Sport and Exercise Settings:*** for example through less mirrors in gyms [71], awareness of visibility, the provision of larger and stronger equipment for all body sizes, less posters and images depicting unrealistic body ideals (e.g. sixpacks), and, as also proposed by Picket and Cunningham [71], by making people with obesity feel comfortable and included in sport and exercise spaces, for instance by using respectful language (this could be a specific goal of gym staff training),
- **Include Playful Fun Activities** rather than designing exercise programmes primarily focussing on weight loss [60, 73].
- ***Anti-Stigma Policies:*** Implement no tolerance policies for any overt discriminatory behaviour (comments, ridicule, marginalisation) in PE classes, sport and exercise settings [71].
- **Educate People with Obesity** about the potentially isolating effects of self-excluding coping strategies.
- **Target Self-Stigma:** Our results show that self-stigma and self-discriminatory attitudes can be barriers to physical activity. Therefore, public health strategies must target the societal roots of self-stigma. As proposed by the research team surrounding Puhl and Schwartz [74] and other experts [46], one important strategy in this regard could be to display positive and motivating images of people with obesity exercising in the media and in sport and exercise settings. The UConn Rudd Center for Food Policy and Obesity website provides a freely accessible media gallery [74].

Finally, weight stigma researchers have urged policymakers to talk to people with obesity directly [57]. We would therefore like to encourage health educators and policy makers to include people with obesity in the design of non-stigmatising sport and exercise settings. The best insights may be gained by asking people with obesity on how to change PA and sport environments.

## Conclusion

To our knowledge, the study presented here is one of the first to provide a detailed account of self-excluding strategies people with obesity employ in sport and exercise settings in order to cope with weight stigma. Our study confirms the assumption that traumatic experiences of being discriminated against, of self-discrimination due to obesity, and of fear of weight stigma can lead to numerous strategies of avoidance and social withdrawal from exercise settings. One important finding of our study, however, is that these coping responses to weight stigma do not necessarily lead to a total self-exclusion from sport and exercise settings. Our results rather indicate that coping responses can be very complex and thus have quite different effects on activity behaviour. The potentially socially isolating effects and consequent impacts on health of such coping strategies should thus be further investigated.

Since there has been a call for more attention to the role of weight stigma specific coping responses and their impact on health behaviours recently [42], future research in this area should analyse exercise-related coping with weight stigma with different sample in more detail. Follow-up quantitative approaches could also examine possible differences in this regard depending on age, gender, social background and life style preferences, which has not been done systematically yet, to our knowledge. Further research on this will be particularly important to determine the characteristics of physical activity programmes for people with obesity that are as discrimination-free as possible.

Finally, it is important to stress that our study counters widespread myths about people with obesity: the results show that they do exercise and also spend a great deal considering how to find ‘safe’, i.e. stigma-free, conditions in order to be active. Based on these findings, and in accordance with other recent works on weight-stigma and physical activity [3, 43, 71], we thus postulate that people with obesity would exercise and participate in sports even more if physical activity spaces were more welcoming and non-stigmatising. Given the great health benefits of regular exercise, it is therefore urgent for public health to focus on fighting the occurrence of weight stigma in exercise settings.

## Supplementary Information

**Additional file 1.**

## Data Availability

The data for this study are confidential. In accordance with received IRB approval, the data is therefore not publicly available in order to guarantee anonymity. Further information about data and conditions for access can be requested from corresponding author.

## Abbreviations

BMI: Body Mass Index
PA: Physical Activity

## Footnotes

1 The authors decided to use a person-first language (“person with obesity”). However, we would like to highlight that the question of how to respectfully use terminology associated with obesity is controversially discussed by stigma experts. Reporting an expert discussion on terminology and best practice from the 2015 Annual International Weight Stigma Conference, Meadows and Daníelsdóttir [2] point out that using the term ‘obesity’ itself is also problematic because it is based on “the medicalization of body state” ( [2] p.2). Scholars have therefore encouraged to use more neutral terms such as ‘higher weight’. Our study was originally designed before we became aware of this. Since the design included a distinction between overweight and obesity based on BMI, we cannot change the term post hoc and opted for a person-first language. Furthermore, the discriminatory experiences we sought to investigate are directly related to the stereotyped term of obesity. This is another reason why we could not avoid the term because the main goal of this study is to explore the stigmatisation of this socially constructed term. We encourage our readers to be critical of established terminology and suggest for further reading: [2, 75].

2 The authors would also like to highlight that ‘sport(s)‘in German has a wider meaning than in (American) English. Sport(s) in German can denote professional or organised sports; yet sport is also widely understood by German speaking people as exercise. For instance, going to the gym, or going for a run is often regarded as ‘sport’ by Germans.
