# Correction to: Weight stigma experiences and self-exclusion from sport and exercise settings among people with obesity

**DOI:** 10.1186/s12889-021-10898-3

**Published:** 2021-05-25

**Authors:** Hendrik K. Thedinga, Roman Zehl, Ansgar Thiel

**Affiliations:** 1 Institute of Sport Science, Eberhard Karls University Tübingen, Tübingen, Germany; 2 Interfaculty Research Institute for Sport and Physical Activity, Eberhard Karls University Tübingen, Tübingen, Germany; 3 Institute of Sport Science, University of Bern, Bern, Switzerland

**Correction to: BMC Public Health 21, 565 (2021)**

**https://doi.org/10.1186/s12889-021-10565-7**

It was highlighted that the original article [1] contained some minor reference and typesetting errors in the Background section. This Correction article shows the incorrect and correct references and citations in the sentences. The original article has been updated.

**Incorrect**

More regularly, “day-to-day discrimination [ …] comes in the form of “micro-aggressions” such as […] misguided comments” ([11] p.n.p.), rude and offensive remarks [9].

**Correct**

More regularly, “day-to-day discrimination […] comes in the form of “micro-aggressions” such as […] misguided comments” ([10] p.n.p.), rude and offensive remarks [9].

**Incorrect**

They regularly encounter “negative weight-related attitudes and beliefs demonstrated by stereotypes, rejection and prejudice” ([16] p.347).

**Correct**

They regularly encounter “negative weight-related attitudes and beliefs that are manifested by stereotypes, rejection and prejudice” ([16] p.347).

Finally, with regard to reference [10], the link provided in the original reference was outdated. The correct URL for reference 10 is: https://www.apa.org/topics/racism-bias-discrimination/types-stress. The reference has been updated.

## References

[1] Thedinga (2021). Weight stigma experiences and self-exclusion from sport and exercise settings among people with obesity. BMC Public Health.

[9] Thiel A, John JM, Carl J, Thedinga HK (2020). Weight stigma experiences and physical (in)activity: a biographical analysis. Obes Facts.

[10] APA. Discrimination, what it is, and how to cope: American Psychological Association; 2019. Available from: https://www.apa.org/topics/racism-bias-discrimination/types-stress.

[11] Lewis S, Thomas SL, Blood RW, Castle DJ, Hyde J, Komesaroff PA (2011). How do obese individuals perceive and respond to the different types of obesity stigma that they encounter in their daily lives? A qualitative study. Soc Sci Med.

[16] Puhl RM, Moss-Racusin CA, Schwartz MB, Brownell KD (2008). Weight stigmatization and bias reduction: perspectives of overweight and obese adults. Health Educ Res.

