# Supplementary material for: Weight stigma experiences and self-exclusion from sport and exercise settings among people with obesity
Source: BMC Public Health. 2021 Mar 22;21:565. doi: 10.1186/s12889-021-10565-7 (PMC7983352; doi:10.1186/s12889-021-10565-7)
Supplement: Supplementary file 1 — Additional file 1. [file 12889_2021_10565_MOESM1_ESM.pdf]

Supplementary Material (Picture / Image of Research) for

Manuscript

Title of Manuscript:

Weight Stigma Experiences and Self-Exclusion from Sport and Exercise Settings among People with Obesity

Hendrik K. Thedinga[1,2], Roman Zehl[3], Ansgar Thiel[1,2,4]

[1] Institute of Sport Science, Eberhard Karls University Tübingen.

[2] Interfaculty Research Institute for Sports and Physical Activity Tübingen, Eberhard Karls University of Tübingen.

[3] Institute of Sport Science, University of Bern, Switzerland.

[4] Orcid ID: https://orcid.org/0000-0001-9217-0442

Corresponding author: Hendrik K. Thedinga, hendrik.thedinga@uni-tuebingen.de.

Info about Supplementary Material:

The supplementary material is an original artwork (sketch). This drawing has been sketched by author Hendrik K. Thedinga in order to give an idea of the study and visualise a key finding of results to reviewers and future potential readers. In case of publication, the image could be used to promote the article, for example on social media outlets. We attached the image in two different sizes and formats.

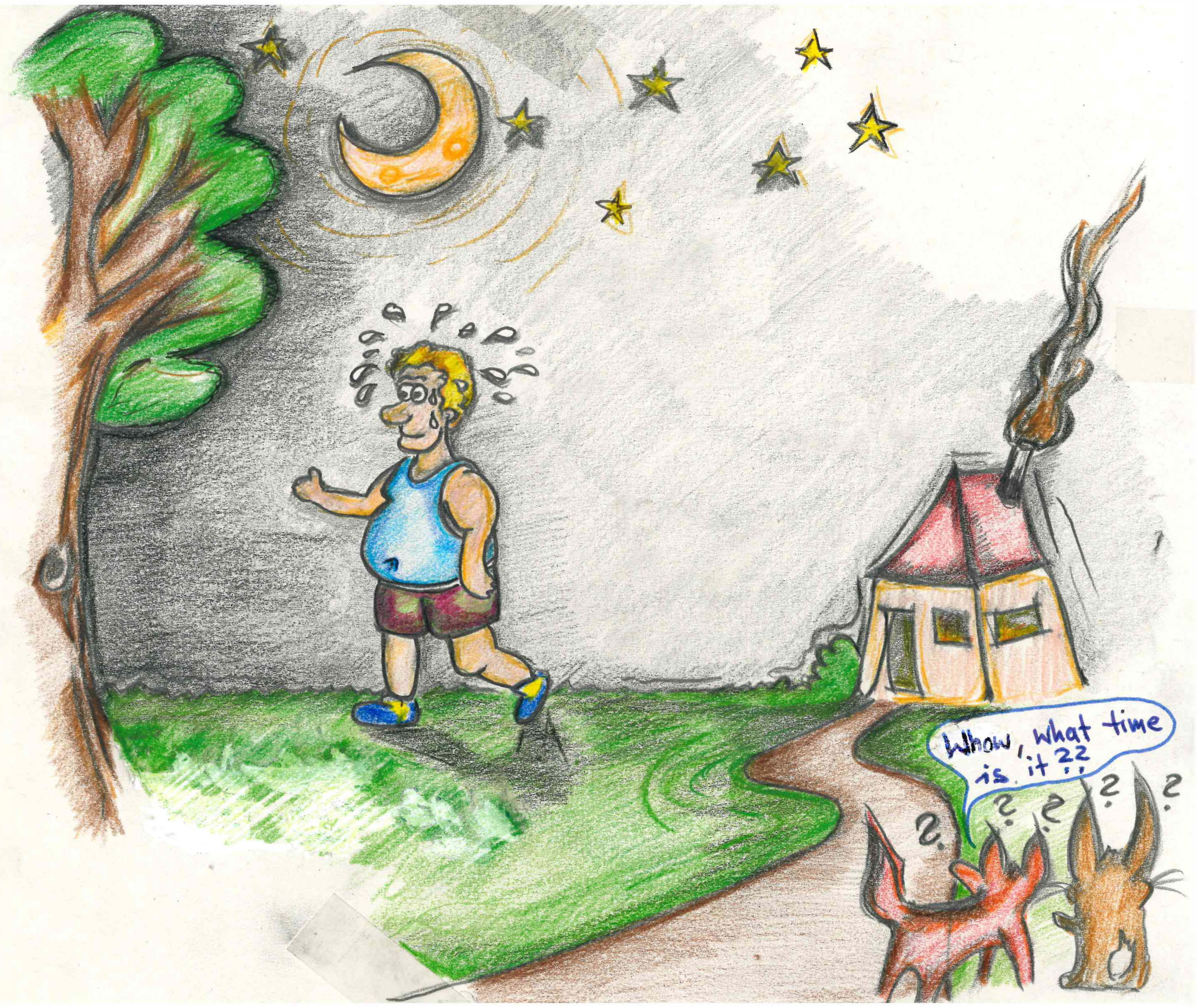
Whow, what time is it ??

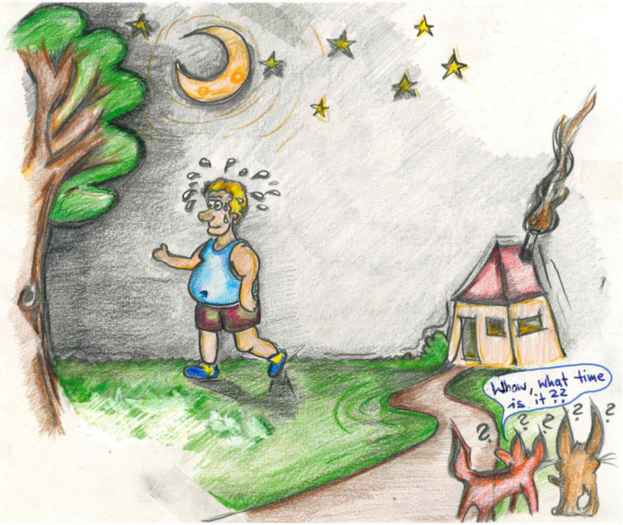
Whow, what time is it ??
